# *SocialViruses*: integrating quantitative phage–bacteria and phage–phage interaction networks for rational cocktail design

**DOI:** 10.1093/bioadv/vbaf239

**Published:** 2025-09-29

**Authors:** Felipe Molina, Manuel Menor-Flores, Miguel A Vega-Rodríguez

**Affiliations:** Departamento de Bioquímica y Biología Molecular y Genética, Universidad de Extremadura, 06006 Badajoz, Spain; Escuela Politécnica, Universidad de Extremadura, 10003 Cáceres, Spain; Escuela Politécnica, Universidad de Extremadura, 10003 Cáceres, Spain

## Abstract

**Motivation:**

Phage therapy is emerging as a promising alternative to antibiotics in biomedical research, highlighting the growing need for computational tools to rationally design effective phage cocktails. However, its clinical potential is often compromised by the evolution of heritable bacterial resistance, which is frequently exacerbated by repeated phage exposure. This can lead to broad-spectrum cross-resistance and reduced long-term efficacy. Existing approaches typically rely on host range matrices but often overlook viral interference and the complex, non-binary nature of virus–host interactions.

**Results:**

We present *SocialViruses*, a tool for designing optimized phage cocktails selecting up to twelve viruses and using two alternative algorithms. *SocialViruses* integrates quantitative host range infection and virus–virus interaction matrices to guide cocktail design. It produces a detailed report with key quality metrics and allows users to define multiple cocktails while minimizing viral interference and managing co-infection redundancy.

**Availability and implementation:**

*SocialViruses* is freely available as a Cytoscape application and can be downloaded from: https://apps.cytoscape.org/apps/SocialViruses.

## 1 Introduction

Failures in phage therapy are common, with the emergence of bacterial resistance widely acknowledged as a significant obstacle (Chan *et al.* 2013). Moreover, repeated exposure to phages can drive the selection of heritable and genetically stable resistance in bacteria, amplifying partial resistance from earlier treatments and resulting in broad cross-resistance that compromises the long-term effectiveness of phage therapy (Kim *et al.* 2024).

From a treatment perspective, there is evidence suggesting that bolus dosing, which involves delivering high concentrations of phages in a single dose, can be more effective than continuous infusion in the early phases of treatment, likely due to faster attainment of therapeutic levels (Smith *et al.* 2023).

The fitness costs of resistance mutations and the ability of viruses to evolve counter-defenses, conform an evolutionary arms race between hosts and viruses (Bohannan and Lenski 2000). A recent model by Rothenburg and Brennan (2020) incorporates the full host-virus interactome to assess viral fitness and host range, determining whether a virus can productively infect and cause disease across different bacterial strains. Interestingly, although evolutionary training may enable viruses to overcome the trade-off between replication and infectivity (Zhang *et al.* 2021), broad host range phages often exhibit reduced virulence (Molina *et al.* 2020).

To mitigate bacterial resistance, phage cocktails—combinations of multiple phages—are frequently used, as the simultaneous evolution of resistance to several phages is considered improbable (Zhao *et al.* 2024). Including at least two phages targeting each host strain has also been proposed to enhance antibacterial efficacy (Abedon *et al.* 2021).

However, results from *in vivo* studies have shown that phage cocktails do not always outperform single-phage treatments. In some cases, cocktails have reduced the virulence (Watanabe *et al.* 2007) or host range (Bourdin *et al.* 2014) of individual phages when targeting pathogens such as *Pseudomonas aeruginosa* or *Escherichia coli*. These findings suggest that antagonistic interactions among phages can diminish overall lytic efficacy (Niu *et al.* 2021). Conversely, synergistic effects have been observed in treatments targeting *Aeromonas salmonicida* (Chen *et al.* 2018), *E. coli* (Schmerer *et al.* 2014), and *P. aeruginosa* (Chaudhry *et al.* 2017).

A significant limitation in most studies designing phage cocktails (Menor-Flores *et al.* 2022) is their dependence on binary host-range categorizations, labeling bacteria simply as “vulnerable” or “non-vulnerable.” Such simplifications ignore key quantitative parameters like infection efficiency, burst size, and latency period, all of which can significantly influence therapeutic outcomes even when a phage might have a broad host range.

Ultimately, the success of phage therapy depends not only on the intrinsic properties of individual phages, such as burst size or adsorption rate, but also on the interactions among phages within a cocktail. These interactions must be carefully considered to design effective combinations. In a previous study analyzing 50 datasets comprising 2877 bacterial strains and 899 phages (Molina *et al.* 2022), cocktail size was not strongly correlated with therapeutic efficacy. In other words, larger cocktails did not necessarily yield better outcomes. Instead, the density of interactions (measured as the ratio of edges to nodes in phage–bacteria interaction networks) was a more reliable predictor of success than the size of the cocktail.

Here, we introduce *SocialViruses*, a novel application that accounts for co-infection by multiple phages and for quantitative interactions both among phages and between phages and their bacterial hosts. By integrating these interaction networks, *SocialViruses* can identify and prioritize phage combinations that are synergistic or neutral, thereby enhancing therapeutic efficacy. Additionally, the application supports the design of multiple, complementary phage cocktails, offering a strategic framework for sustained and adaptive phage therapy.

## 2 Results

**Figure 1.**
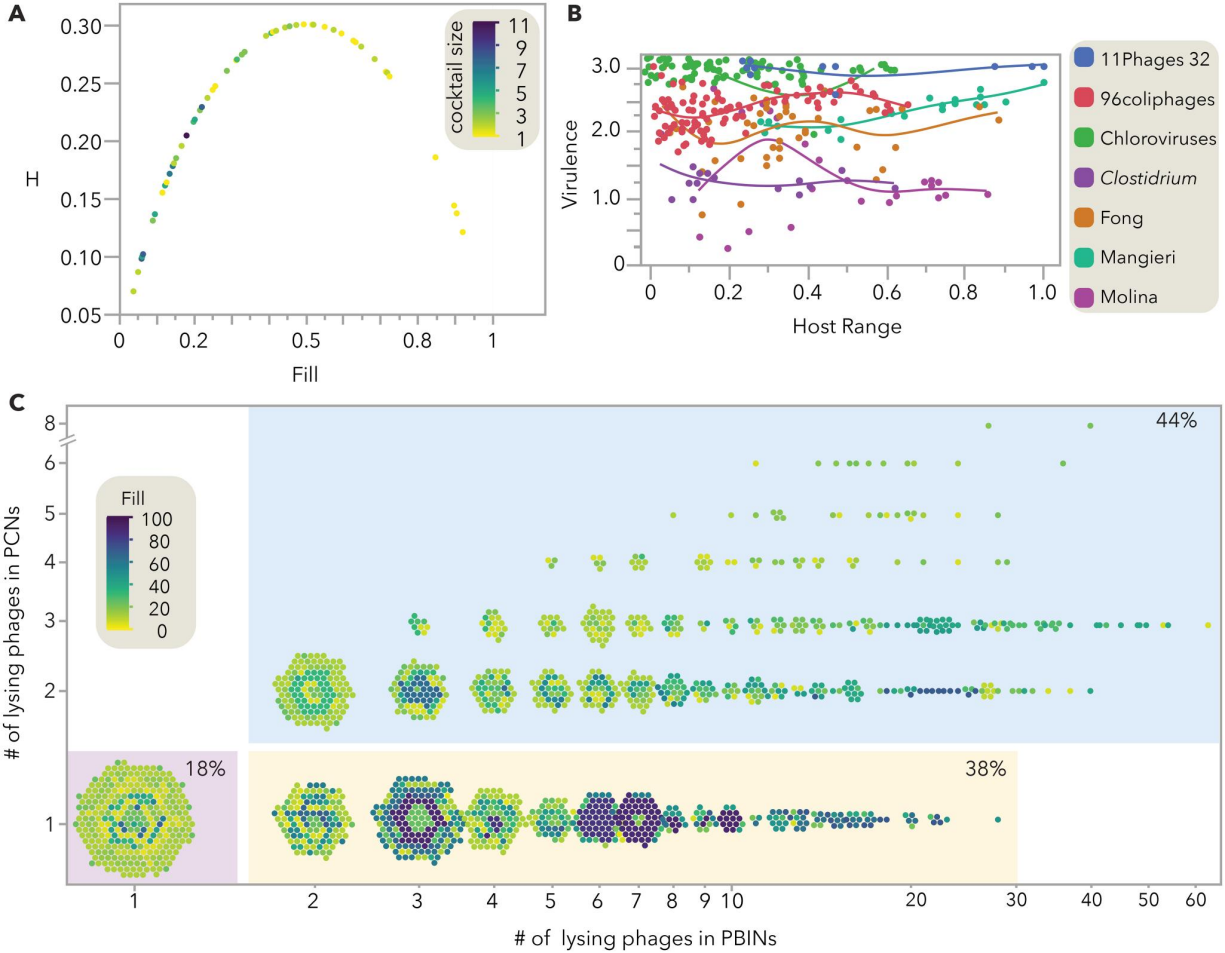
Analysis of phage bacteria infection networks. (A) Entropy (H) values from 50 datasets comprising 2877 bacterial strains and 899 phages (Molina *et al.* 2022). For each matrix, the fill and estimated cocktail size are shown. (B) Virulence and host range of individual phages from seven datasets were calculated as described in Molina *et al.* (2020). Virulence values range from 0 (no infection) to 3 (complete inhibition of bacterial growth), while host range values range from 0 (no bacterial host infected) to 1 (all bacterial strains infected). Smooth lines were fitted using a cubic spline with a lambda of 0.05. (C) Number of lysing phages in the 50 datasets (PBINs)and the resulting cocktails (PCNs) generated by *PhageCocktail* (Molina *et al.* 2022). Each dot represents a bacterial host, with color indicating the fill (%) of the PBIN.

In a previous study, we developed *PhageCocktail* (Menor-Flores *et al.* 2022), a tool designed to generate phage cocktails based solely on binary phage–bacteria infection networks (PBINs). To assess the potential benefits of incorporating quantitative phage–bacteria and phage–phage interactions, we analyzed the structure of experimental PBINs (Fig. 1).

### 2.1 Datasets

Datasets were sourced from a meta-analysis (Molina *et al.* 2022), which includes matrices ranging from 21 to 12 450 entries and features bacteria and phages from diverse environments such as seafood, plants, livestock, dairy, sewage, and clinical isolates. Additionally, virulence and host range (Fig. 1B) were analyzed using seven datasets: the first four downloaded from the Viral Host Range database (https://viralhostrangedb.pasteur.cloud; Lamy-Besnier *et al.* 2021), and the remaining three from the meta-analysis.

### 2.2 Network-based insights into phage cocktail design: entropy, virulence, and interaction dynamics

Binary entropy (H), which reflects the combinatorial complexity, was calculated, for the 50 datasets (Fig. 1A) using matrix fill (Strydom *et al.* 2021). Although both highly and sparsely filled matrices exhibit lower combinatorial complexity, only the former clearly require a small number of phages to lyse the bacterial hosts. Notably, these highly filled matrices are rare due to the high specificity phage predation on bacteria, and therefore most cocktails cannot be easily designed through visual approximation.

Although host range expansion may be advantageous for phages, generalism can involve trade-offs, such as reduced propagation rate (Ford *et al.* 2014). To compare growth inhibition intensity (virulence) and host range amplitude, quantitative data from a few available QPBINs was analyzed (Fig. 1B) as described by Molina *et al.* (2020). In most QPBINs, phages with similar host ranges exhibit varying levels of virulence. In some datasets, virulence peaked at intermediate host range values, but the pattern was inconsistent, suggesting that virulence should be evaluated individually for each QPBIN.

Finally, the frequence of putative phage–phage interactions (Fig. 1C) was examined in complete PBINs and in the trimmed Phage Cocktail Networks (PCNs). In cocktails, a maximum of eight phages infected the same host, whereas is the untrimmed networks several bacterial hosts are infecting by >50 different phages. Notably, only 18% of the 2877 bacterial strains were targeted by a single phage, while 44% were targeted by two or more phages in the designed cocktails. The remaining strains were infected by multiple phages in the PBINs but only by one in the cocktails. Interestingly, the variation of redundancy in phage interactions between PBINs and PCNs was higher in highly filled matrices than in sparsely filled ones. These results indicate that constructing quantitative phage–phage interaction networks (QPPINs) for subsets of each phage collection may enhance cocktail performance.

## 3 Implementation and features

### 3.1 Impact of quantitative interaction networks on phage cocktail optimization

**Figure 2.**
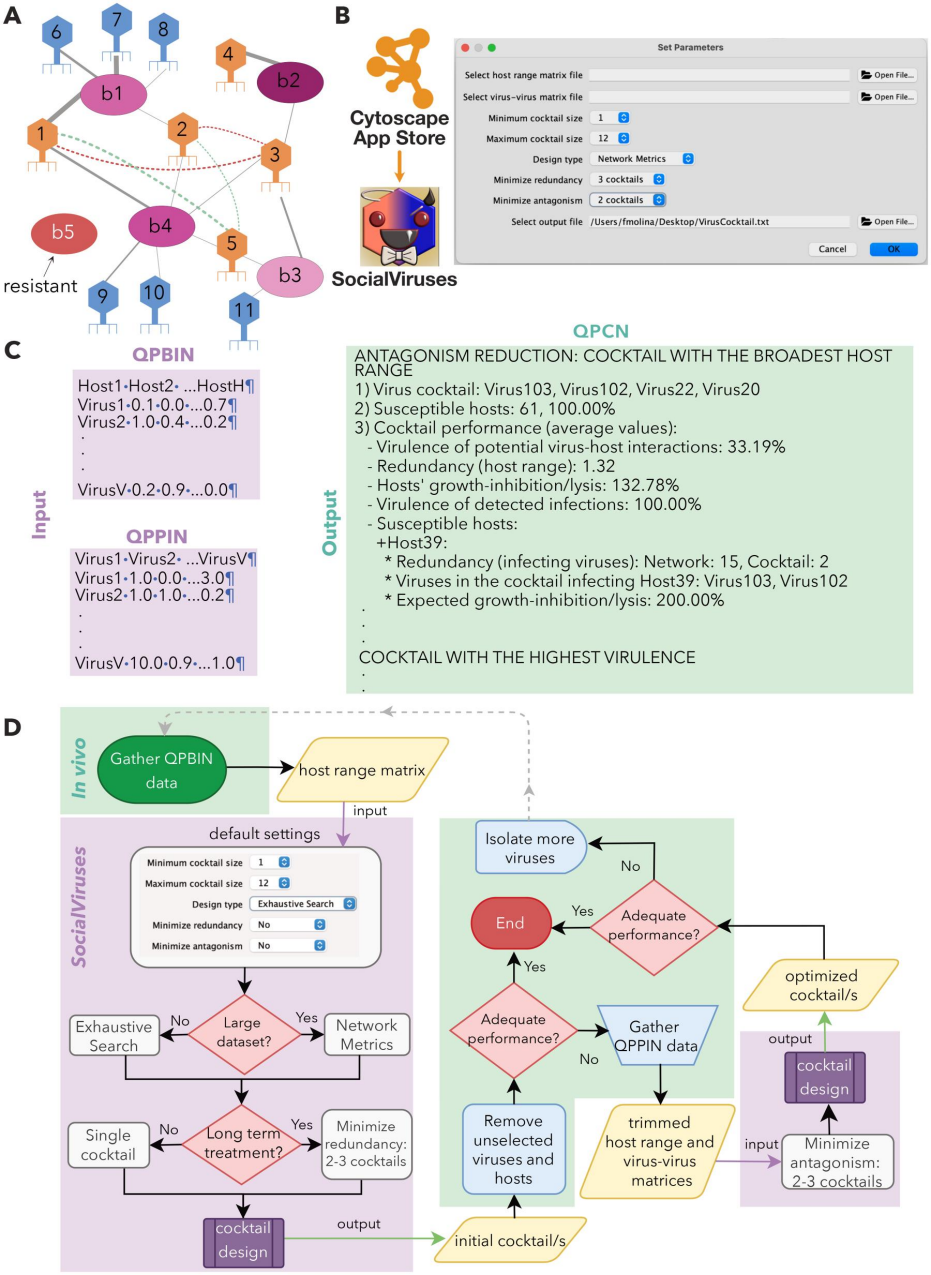
SocialViruses: interface, data structure, and workflow. (A) Examples of quantitative phage–bacteria and phage–phage interactions. Bacteria are represented by ellipses. Solid lines indicate phage–bacteria interactions, while dotted lines represent phage–phage interactions. Line width reflects interaction strength, and color denotes interaction type (grey: infection, red: antagonism, green: synergy). (B) Installation and graphical user interface of *SocialViruses*, showing options for loading input matrices, selecting the algorithm, and configuring design parameters (size, redundancy, and antagonism). (C) Examples of input and output files. Input QPBIN matrices represent quantitative virus–host interactions, with rows corresponding to viruses and columns to hosts. Input QPPIN capture quantitative virus-virus interaction. An output file illustrating two cocktail design strategies: one that minimizes antagonism by selecting viruses with broad host ranges, and another that maximizes virulence. Performance metrics include host susceptibility, virulence, redundancy, and predicted growth inhibition or lysis (D). Overview of a proposed workflow for phage cocktail design and optimization, integrating both *in vivo* (green) and *in silico* (purple) components. It begins with QPBIN data, which can be generated through cross-streak assays (Molina *et al.* 2020), followed by the design of an initial set of cocktails to filter and refine the dataset. Based on treatment goals (minimizing antagonism or maximizing virulence), and the performance of the initial cocktails, refinement strategies are applied. These may include QPPIN data, obtained through growth curve analysis (Niu *et al.* 2021), or isolating additional viruses.

Unlike *PhageCocktail*, which is limited to binary PBINs, *SocialViruses* supports both Quantitative phage–bacteria infection networks (QPBINs) and QPPINs. This broader input allows *SocialViruses* to factor in phage virulence and phage–phage interactions during cocktail design. In contrast, *PhageCocktail* classifies phages solely based on their host range. As a result, while *SocialViruses* assigns distinct scores to phages infecting the same host (e.g. host *b1* in Fig. 2A, where phage 1 ranks highest), *PhageCocktail* cannot differentiate between phages with identical host ranges, such as phages 1 and 2 or phages 6, 7, and 8. By leveraging quantitative data, *SocialViruses* selects an optimal cocktail (phages 1, 4, and 5), avoiding antagonistic effects from phage 3 and favoring phage 1 over phage 2. In contrast, *PhageCocktail* incorrectly designs a minimum-sized cocktail that includes phage 3 and either phage 1 or 2.

### 3.2 Installation and user interface

To install *SocialViruses*, users must first install Cytoscape 3.x, then navigate to Apps > Show App Store and search for “SocialViruses.” The application features a user-friendly interface for automated analysis and requires a host range matrix as input, with an optional virus–virus interaction matrix (Fig. 2B). Users can proceed with default settings or customize parameters such as cocktail size, design type, and constraints. *SocialViruses* also enables minimization of phage antagonism and redundancy during cocktail design, enhancing both effectiveness and efficiency. The resulting phage cocktails are exported for downstream use, and a Phage Cocktail Network (PCN) is generated for visualization and further analysis within Cytoscape.

To mitigate risks such horizontal gene transfer, dysbiosis, or high production costs, phage cocktails are limited to a maximum of 12 phages (adjustable by the user). The application aims to identify the smallest cocktail capable of lysing the largest number of bacteria using two methods: Network Metrics and Exhaustive Search as implemented in the *PhageCocktail* app (Menor-Flores *et al.* 2022).

Network Metrics uses a computationally efficient heuristic that prioritizes phages based on host range breadth and the resistance profiles of bacterial host on their resistance, making it well-suited for exploratory analyses or large datasets. In contrast, Exhaustive Search systematically evaluates all possible phage combinations up to a user-defined size limit. Although more computationally intensive, it guarantees optimal solutions under specified constraints and is best suited for smaller datasets or when precision is essential.

### 3.3 Data structure: input and output files

QPBIN input files represent matrices M = (V, H), where V denotes viruses and H denotes bacterial hosts (Fig. 2C). The first row lists host names (e.g. Host1, Host2) while the first column (excluding the top-left cell) lists phage names (e.g. Virus1, Virus2, etc.). Each cell contains a quantitative measure of infection strength or virulence, with values ranging from 0 (no lysis) to 1 (complete lysis). For ease of input, data entries are accepted in plain text format, with columns separated by spaces and rows delimited by line breaks.

Similarly, QPPIN files correspond to square matrices. A value of 1 indicates a neutral or unknown interaction. Antagonistic interactions are represented by values <1, with 0 indicating complete lethality, while synergistic interactions are indicated by values >1.

The structure of the output file varies depending on the selected settings and design constraints (Fig. 2C). It includes configuration parameters, corrections applied to the input matrices, and performance metrics for Phage Cocktail Networks (PCNs) of varying sizes. For each cocktail size, the file displays the PCNs that achieved the broadest host range or the highest virulence. PCNs are evaluated on several key criteria: number of susceptible hosts, infection virulence, redundancy, and predicted host growth inhibition. Additionally, the output file can incorporate alternative cocktails optimized to reduce antagonistic interactions among phages. Each PCN includes a detailed breakdown of which viruses target which hosts, the expected lysis efficiency, and the individual contribution of each virus to the overall performance. This structured output enables users to compare and select phage combinations based on specific therapeutic goals such as broad-spectrum coverage, high potency, or minimal redundancy.

### 3.4 A Streamlined workflow for phage cocktail optimization

Given that constructing QPPINs is both time- and resource-intensive, a streamlined workflow for optimizing phage cocktail design is proposed (Fig. 2D). Thus, the initial cocktail design relies on a QPBIN dataset, with Exhaustive Search recommended to identify optimal combinations. To mitigate the emergence of resistance mutations in host bacteria, using multiple cocktails is advisable for long-term treatment. If *in vivo* evaluation of the generated cocktails reveals suboptimal performance, it becomes necessary to construct the QPPIN from the subset of phages comprising the initial PCNs. Subsequently, a new set of PCNs that minimize antagonistic interactions can be generated by *SocialViruses*. Finally, if the performance of the optimized cocktails remains inadequate, the inclusion of additional phages should be considered.

## 4 Discussion and future directions

*SocialViruses* represents a significant advancement in the rational design of phage cocktails, addressing key limitations in current phage therapy strategies (Table 1). Previous approaches have often relied on global properties of PBINs (Díaz-Galián *et al.* 2022) or on binary host-range matrices (Menor-Flores *et al.* 2022), which tend to oversimplify the complex and dynamic nature of phage–bacteria interactions. For instance, it has been observed that phage virulence may decrease as host range increases (Molina *et al.* 2020), highlighting the need for more nuanced modeling.

**Table 1.** Comparative overview of *SocialViruses*, *PhageCocktail*, and digital phagograms (Lood *et al.* 2022).

Feature	*SocialViruses*	*PhageCocktail*	Digital phagograms
Available for the user	Yes	Yes	No
Interaction type	Quantitative Phage–Bacteria (QPBIN) and Phage–Phage (QPPIN) networks	Binary Phage–Bacteria Infection Networks (PBINs)	Multilayer ML model integrating genomic, proteomic, and transcriptomic data
Phage selection criteria	Virulence, host range, redundancy, antagonism/synergy	Host range only (binary infection)	Predictive infectivity based on omics features and layered biological processes
Cocktail optimization	Yes	Not directly for cocktail design	Not directly for cocktail design
Redundancy management	Minimizes co-infection redundancy	Ignored	Not explicitly addressed
Antagonistic interactions	Avoids antagonistic phage–phage interactions	Not considered	Not modeled directly, but could be inferred from interaction layers
Synergistic effects	Can prioritize synergistic combinations	Not detected	Potentially inferred from multi-layer predictions
Data requirements	Quantitative infection and interaction matrices. No minimum sample size required.	Binary infection data. No minimum sample size required	High-quality multi-omics data (genomics, proteomics, transcriptomics). Minimum sample size.
Customization	Adjustable cocktail size, design goals, constraints	Limited customization	Customizable ML layers and feature sets per species
Output detail	Detailed metrics: host susceptibility, virulence, redundancy, lysis prediction	Basic host coverage	Layered infectivity predictions per phage-host pair
Adaptability	Iterative refinement based on *in vivo* results	Static design	Adaptive learning from accumulating data
Limitations	Requires high-quality quantitative data; QPPINs are resource-intensive	Oversimplifies interactions; ignores virulence variability	Requires extensive annotated datasets; interpretability versus accuracy trade-offs

By integrating quantitative QPBINs and QPPINs, *SocialVirus*es offers a comprehensive framework for phage cocktail design. Its ability to minimize viral antagonism and coinfection redundancy significantly enhances therapeutic efficacy. The tiered workflow, starting with QPBINs and incorporating QPPINs only when needed, is especially valuable in clinical and ecological settings with limited resources. This adaptive strategy enables iterative refinement based on *in vivo* outcomes. By supporting the design of cocktails that reduce resistance and antagonism, *SocialViruses* aligns with public health efforts to curb antibiotic use and enables personalized phage therapy tailored to specific bacterial profiles and treatment goals.

Nevertheless, the effectiveness of *SocialViruses* relies heavily on the quality and completeness of the input data. To ensure reliability, conducting biological experiments in triplicate is recommended (Molina *et al.* 2020). Unlike evolutionary (Beckett and Williams 2013) and ecological (Torres‐Barceló *et al.* 2025) studies, clinical studies (Molina *et al.* 2022) often simplify networks by excluding narrow host-range phages, thereby removing specialists that may exhibit high virulence. Constructing accurate QPBINs and QPPINs requires substantial experimental effort, and the lack of standardized datasets may limit its immediate applicability across diverse microbial systems. On the other hand, the genomic similarity between different phages or hosts (Wei *et al.* 2024) could be used for the *in silico* generation of these networks.

Future improvements might include integrating machine learning models trained on empirical data to predict interaction outcomes (Lood *et al.* 2022). However, the proposed three-layer model faces key limitations: it requires diverse datasets, struggles to integrate predictions across layers, and is hindered by data sparsity, particularly for poorly characterized phages or hosts. In contrast, *SocialViruses* performs effectively from the outset, without relying on prior dataset training. Overall, *SocialViruses* provides a robust and adaptable framework for designing phage cocktails based on quantitative interaction data.

## Software availability

The software  is freely available as a Cytoscape application and can be downloaded from: https://apps.cytoscape.org/apps/SocialViruses.
